# Updated insights into the NLRP3 inflammasome in postoperative cognitive dysfunction: emerging mechanisms and treatments

**DOI:** 10.3389/fnagi.2024.1480502

**Published:** 2024-09-30

**Authors:** Tian Wang, Guangwei Sun, Bingdong Tao

**Affiliations:** Department of Anesthesiology, Shengjing Hospital of China Medical University, Shenyang, China

**Keywords:** POCD, neuroinflammation, NLRP3, inflammasomes, pyroptosis, autophagy POCD, autophagy

## Abstract

Postoperative cognitive dysfunction (POCD) poses a significant threat to patients undergoing anesthesia and surgery, particularly elderly patients. It is characterized by diminished cognitive functions post surgery, such as impaired memory and decreased concentration. The potential risk factors for POCD include age, surgical trauma, anesthetic type, and overall health condition; however, the precise mechanisms underlying POCD remain elusive. Recent studies suggest that neuroinflammation might be a primary pathogenic factor. NOD-, LRR- and pyrin domain-containing protein 3 (NLRP3) inflammasomes are implicated in exacerbating POCD by promoting the release of inflammatory factors and proteins that initiate pyroptosis, further influencing the disease process. The regulation of NLRP3 inflammasome activity, including its activation and degradation, is tightly controlled through multiple pathways and mechanisms. In addition, autophagy, a protective mechanism, regulates the NLRP3 inflammasome to control the progression of POCD. This review reviews recent findings on the role of the NLRP3 inflammasome in POCD pathogenesis and discusses therapeutic strategies aimed at reducing NLRP3 sources, inhibiting cellular pyroptosis, and enhancing autophagy.

## 1 Introduction

Postoperative cognitive dysfunction (POCD) represents a significant neurological complication characterized by declines in learning, memory, attention, language, and executive functions and can persist for days to years (Nabil et al., 2023). The incidence of POCD following noncardiac surgery varies significantly, ranging from 2.2 to 31.5%, and that following cardiac surgery varies from 11.8 to 35.7% (Arefayne et al., 2023). Risk factors include advanced age, high levels of neuroinflammatory mediators in the blood, reduced oxygen saturation (SpO2), prolonged anesthesia, extensive surgery duration, and deep anesthesia (Arefayne et al., 2023). Alarmingly, POCD is associated with increased mortality, early workforce exit, and the onset of new disabilities, imposing substantial burdens on individuals, families, and societal resources (Peng et al., 2023). This underscores the critical need to investigate the mechanisms of POCD and identify effective interventions.

Although the precise mechanisms underlying POCD remain elusive and robust preventative and therapeutic measures are lacking, current research has identified several potential contributors, including neuroinflammation (Safavynia and Goldstein, 2019), oxidative stress (Netto et al., 2018; Liu Q. et al., 2021), hormonal imbalances (Wang et al., 2019), neurotransmitter disruptions (Wang et al., 2019), and anesthetic toxicity (Dong et al., 2022). Among these, neuroinflammation is particularly significant in the occurrence of age-related postoperative cognitive dysfunction (Safavynia and Goldstein, 2019; Li et al., 2022), with treatment strategies aimed at mitigating neuroinflammatory responses showing promise. Fundamentally, inflammation is an immune response to injury, infection, or stress that activates immune cells and releases cytokines such as TNF-*α*, IL-1β, and IL-6 (Christgen et al., 2020). Microglia, the resident immune macrophages in the brain, are essential for maintaining CNS homeostasis and defending against pathogens (Butovsky and Weiner, 2018). There is significant evidence that mice with POCD exhibit abnormal activation of microglia in the hippocampus, where the inflammatory response is mediated by the inflammasome (Lin et al., 2024; Wang et al., 2018; Yang et al., 2024).

The inflammasome, a cytosolic multiprotein complex, plays a crucial role in the innate immune response to infection and tissue injury (Yao et al., 2024). It consists of a pattern-recognition receptor (PRR), the adaptor apoptosis-associated speck-like protein containing a CARD (ASC), and an effector known as caspase-1 (Yao et al., 2024; Platnich and Muruve, 2019). The PRR detects exogenous or endogenous signals, the adaptor ASC helps assemble inflammasomes, and caspase-1 produces inflammatory factors that induce inflammation (Zhan et al., 2023). Various types of inflammasomes, such as NLRP1, NLRP3, NLRC4, and AIM2, exist, depending on the sensor (Zhang et al., 2021). However, only the NLRP3 inflammasome has been extensively studied in relation to POCD because it can be activated by diverse triggers, including pathogen- and danger-associated molecular patterns (Wang et al., 2018; Cho et al., 2022). Upon sensing these triggers, the NLRP3 inflammasome promotes the release of the proinflammatory cytokines IL-1β and IL-18 and induces pyroptosis via GSDMD (Shi et al., 2017; Zhao et al., 2019). Pyroptosis, a form of programmed cell death triggered by the inflammasome, has been particularly noted for its role in microglial pyroptosis induced by the NLRP3 inflammasome pathway in aged mice with POCD (Lin et al., 2024). Additionally, NLRP3 inflammasome activation is accompanied by autophagy, a cellular defense strategy that degrades ingested substances, including misfolded proteins and damaged mitochondria, to maintain cellular function and homeostasis (Gordon et al., 2018). Emerging evidence suggests that the inhibition of autophagy exacerbates the extent of NLRP3 inflammasome-mediated inflammation and neurodegenerative diseases (Yuan et al., 2018).

In this review, we detail the mechanisms and roles of NLRP3 inflammasome activation, pyroptosis, and autophagy in the pathogenesis of POCD. We also discuss therapeutic strategies to reduce the sources of NLRP3, inhibit pyroptosis, and enhance autophagy.

## 2 NLRP3 inflammasome: a key mediator in POCD

The involvement of the NLRP3 inflammasome in POCD triggered by anesthesia and surgery has been substantiated through extensive research. Various studies have explored this connection using models developed either with anesthesia alone or in combination with surgical procedures. For instance, exposure to sevoflurane has been shown to alter the levels of serum metabolites, such as isatin, in both elderly patients and aging mice. This alteration promotes microglial proliferation and upregulates proinflammatory gene expression, thereby exacerbating hippocampal inflammation (Wang T. et al., 2024). Similarly, Zhang et al. used isoflurane to establish a POCD model in aged rats, and demonstrated that reducing NLRP3 expression via lentivirus-specific short hairpin RNA mitigated microglial activation and the expression of inflammatory markers including NLRP3, ASC, caspase-1, IL-18, and IL-1β (Zhang et al., 2020). These findings are complemented by observations that the administration of halothane drugs elevated the levels of these inflammatory substances (Zhang et al., 2020). Further research by Liu et al. revealed that repeated exposure to propofol induced neuronal damage and cognitive impairment in aged rats, accompanied by an increase in NLRP3 and caspase-1 in the hippocampus (Liu P. et al., 2021). Notably, pretreatment with Bay-11-7082, an inhibitor of both the NLRP3 inflammasome and NF-κB, reversed these effects, highlighting the contributory role of the NLRP3 inflammasome in anesthesia-induced POCD (Liu P. et al., 2021). Additionally, propofol directly causes pyroptosis of microglia, which is dependent on caspase-1, via the NLRP3-ASC inflammasome mechanism (Sun et al., 2019). Long et al. further established a POCD model through partial hepatectomy surgery under isoflurane anesthesia in 18-month-old mice (Sun et al., 2022). The affected mice displayed notable cognitive deficits in the Morris water maze (MWM) tests, such as prolonged escape times and reduced time spent in the target quadrant, indicating significant memory, and learning impairments. These cognitive deficits were associated with significant increases in the levels of NLRP3, ASC, caspase-1, phosphorylated IκB-*α*, and p65 levels in the hippocampus, emphasizing the pivotal role of the NLRP3 inflammasome in both surgery and anesthesia-induced POCD. Consequently, delineating the pathway mechanisms of the NLRP3 inflammasome is essential for developing targeted strategies to mitigate and prevent POCD (Sun et al., 2022). Consequently, delineating the pathway mechanisms of the NLRP3 inflammasome is essential for developing targeted strategies to mitigate and prevent POCD.

## 3 The mechanism of the NLRP3 inflammasome in POCD

The NLRP3 inflammasome pathway involves a complex interplay of factors and is regulated by numerous substances. Activators of NLRP3 include a variety of viral and bacterial proteins (e.g., nigericin and viroporins), and intracellular molecules released into the extracellular fluid (e.g., extracellular ATP) (Christgen et al., 2020; Mangan et al., 2018). Upon detection of these activators, the NACHT domain of the NLRP3 protein binds to ATP and NIMA-associated kinase 7 (NEK7). This interaction leads to the oligomerization of NLRP3 and the exposure of the PYD domain. These structural changes facilitate the recruitment of the adaptor protein apoptosis-associated speck-like protein containing a CARD (ASC) and the zymogen caspase-1, initiating the assembly of the inflammasome (Christgen et al., 2020). The activation of pro-caspase-1 leads to the cleavage of gasdermin D (GSDMD) into its N-terminal and C-terminal domains (GSDMD-N and GSDMD-C). Activated caspase-1 also processes pro-IL-1β and pro-IL-18 into their mature forms (Lawlor and Vince, 2014; Swanson et al., 2019). GSDMD-N then forms pores in the plasma membrane, facilitating the release of IL-1β and IL-18 and triggering pyroptosis, a form of programmed cell death (Li et al., 2024). These processes underscore the activation of the NLRP3 inflammasome and the subsequent induction of pyroptosis. This paper focuses exclusively on the primary inflammasome activation pathways that trigger pyroptosis—the canonical pathway.

### 3.1 Canonical NLRP3 inflammasome activation pathway

**Figure 1:**
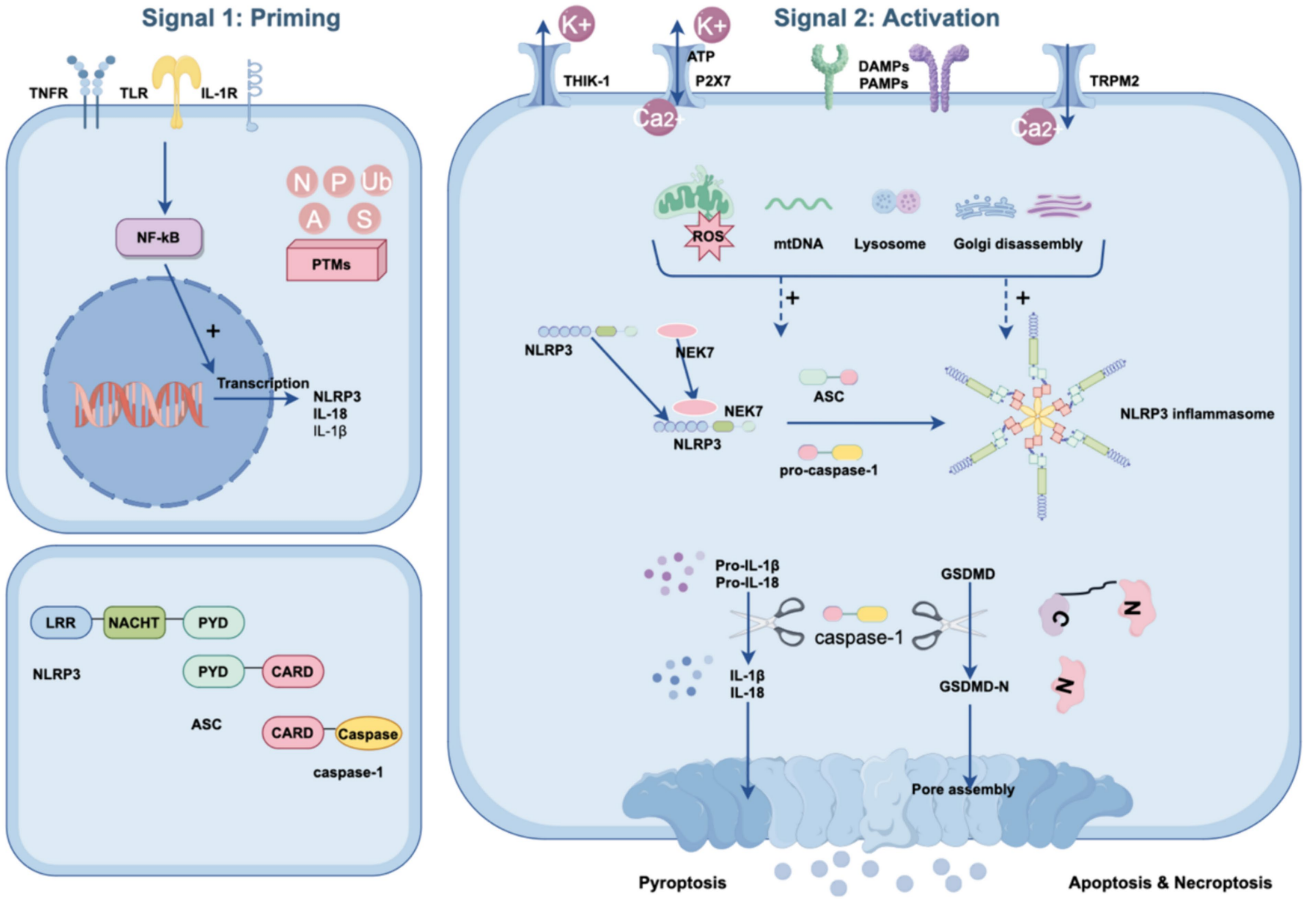
Canonical NLRP3 inflammasome signaling pathway. The canonical NLRP3 inflammasome pathway requires two steps: the priming step and the activation step. During the priming step, stimuli are sensed by TLRs, IL-1Rs, and TNFRs, inducing the activation of NF-κB to migrate into the nucleus. Subsequently, NF-κB mediates the expression of pro-IL-1b, pro-IL-18 and NLRP3. During the activation step, numerous PAMPs or DAMPs promote NLRP3 inflammasome assembly via ASC and pro-caspase-1. Simultaneously, ATP and NEK7 also assist in the assembly process, in which ATP and NEK7 bind to the NACHT domain of NLRP3, resulting in NLRP3 oligomerization and PYD exposure, respectively. These changes lead to the activation of pro-caspase-1 and the cleavage of gasdermin D. Activated caspase-1 also converts pro-IL-1β and pro-IL-18 to their mature forms. The N-terminal domain of GSDMD forms pores in the plasma membrane with the release of mature inflammatory cytokines and pyroptosis. In addition, PTMs of the NLRP3 inflammasome, such as ubiquitination (Ub), phosphorylation (P), alkylation (A), sumoylation (S), and S-nitrosylation (N), can occur at any step in the pyroptosis pathway to facilitate its activation.

Canonical activation of the NLRP3 inflammasome consists of two primary steps: priming and activation (Huang et al., 2021) (see Figure 1).

#### 3.1.1 The priming of NLRP3

The priming step serves dual functions. The first is to upregulate the transcription of the NLRP3 inflammasome components NLRP3, pro-IL-18, and pro-IL-1β. This transcriptional regulation is initiated by the activation of receptors such as the tumor necrosis factor receptor (TNFR), Toll-like receptors (TLRs), and the interleukin 1 receptor (IL-1R) through their respective ligands. These interactions promote the translocation of NF-κB into the nucleus, triggering the transcription of these components (Moretti et al., 2022; Que et al., 2024). Second, the priming step induces multiple posttranslational modifications (PTMs) of NLRP3, such as deubiquitination, acetylation, SUMOylation, and phosphorylation, which licenses NLRP3 for subsequent activation (Que et al., 2024; Zhang S. et al., 2023). For example, NLRP3 is typically maintained in a ubiquitinated state, which inhibits its ability to oligomerize and undergo subcellular translocation (Lopez-Castejon, 2020). Exposure to lipopolysaccharide (LPS) reduces ubiquitin-mediated proteasomal processing of NLRP3 by increasing the levels of an E3 ligase component, FBXO3, which targets FBXL2. FBXL2 then interacts with NLRP3 at Trp-73 and targets Lys689 for ubiquitin ligation and degradation (Xu et al., 2023; Han et al., 2015). Taken together, priming is critical for the activation of the NLRP3 inflammasome, and therefore, modulating specific processes during the priming phase offers a potential strategy for combating POCD.

#### 3.1.2 The activation of NLRP3

During the activation phase, NLRP3 can be activated and initiate self-oligomerization to form a multiprotein inflammasome complex. This activation occurs upon the recognition of diverse signals from various pathogens, including bacteria, viruses, fungi, and crystals, as well as endogenous stressors or aggregated substances. The intricacies of NLRP3 activation are influenced by multiple upstream events, which may act in tandem or independently. These events include ion fluxes, such as potassium efflux (Yaron et al., 2015), calcium efflux (Murakami et al., 2012; Uryash et al., 2024), and chloride efflux (Swanson et al., 2019; Green et al., 2018), and organelle damage, including lysosomal rupture (Hornung et al., 2008), mitochondrial dysfunction, and Golgi disintegration (Chen and Chen, 2018). Importantly, NIMA-associated kinase 7 (NEK7), a kinase involved in mitosis, has been identified as a critical requirement for the activation of the NLRP3 inflammasome (Sharif et al., 2019).

### 3.2 The upstream event of NLRP3 activation: ion flux

Ion flux plays a pivotal role in the activation of the NLRP3 inflammasome, and involves various ion channels that mediate critical ionic movements. For instance, the ATP-gated ion channel P2X purinoceptor 7 (P2X7) facilitates K+ efflux and Ca2+ influx, disrupting mitochondrial ion balance. This disruption generates mitochondrial reactive oxygen species (mROS), triggering NLRP3 inflammasome activation (Di Virgilio et al., 2017; Kong et al., 2022; Wang et al., 2020). Similarly, two-pore domain potassium (K2P) channels, such as ATP-induced two-pore domain weak inwardly rectifying K+ channel 2 (TWIK2) and THIK-1, enhance K+ efflux, which further facilitates NLRP3 activation (Wu et al., 2022; Di et al., 2018). Notably, THIK-1 is predominantly expressed in human microglia, suggesting that targeting this channel could be a viable approach for treating neuroinflammation (Rifat et al., 2024). Additionally, K+ efflux is essential for the activation of the NLRP3 inflammasome through the binding of NEK7 to NLRP3, a critical step in the activation process (Groß et al., 2016).

Another significant channel involved is the transient receptor potential melastatin type 2 (TRPM2), which is highly expressed in the brain. As a nonselective Ca2 + −permeable channel. TRPM2 exacerbates neuroinflammation and cognitive deficits through its role in NLRP3 inflammasome activation (Shao et al., 2021). This activation is largely due to increased intracellular Ca2+ concentration, which facilitates ASC recruitment to NLRP3 (Lee et al., 2012), leading to mitochondrial overload, damage, and subsequent reactive oxygen species (ROS) production (Murakami et al., 2012; Groslambert and Py, 2018), thereby promoting NLRP3 inflammasome activation.

### 3.3 The upstream event of the NLRP3 activation: organelle dysfunction

Dysregulation of various organelles is considered a significant contributor to POCD by facilitating the activation of the NLRP3 inflammasome. This encompasses mitochondrial dysfunction, disturbances in lysosomes, and disintegration of the trans-Golgi apparatus.

#### 3.3.1 Mitochondrial dysfunction and oxidative stress

Exposure to anesthetics and surgical procedures can compromise mitochondrial function, enhancing oxidative damage to neurons (Netto et al., 2018; Skvarc et al., 2018). Mitochondria function as both the primary sites of reactive oxygen species (ROS) production and targets for ROS-mediated damage (Cedikova et al., 2016; Galley, 2011). The interaction between damaged mitochondrial DNA (mtDNA) and ROS with the NLRP3 inflammasome triggers an inflammatory response (Kelley et al., 2019). Reactive oxygen species (ROS) act as crucial activators of the NLRP3 inflammasome, leading to the activation of inflammatory cascades and cellular damage through the ROS-NLRP3 pathway. Evidence of oxidative stress can be viewed as a series of subsequent inflammation-related responses produced by an excess of reactive oxygen species (ROS). At appropriate cellular concentrations, reactive oxygen species (ROS) can act as “REDOX messengers” and play an important role in intracellular signaling and regulation. Reactive oxygen species (ROS) are one of the key factors in the activation of NLRP3 inflammasome. Oxidative stress leads to activation of an inflammatory cascade and cell damage through the NLRP3 pathway via reactive oxygen species (ROS). In sevoflurane-induced rat models of POCD, the accumulation of reactive oxygen species (ROS), along with increased expression of GSDMD-N, cleaved caspase-1, ASC, IL-1β, and IL-18, has been demonstrated (Zhou Y. et al., 2023). Remarkably, these alterations can be mitigated by the administration of N-acetylcysteine (NAC) (Zhou Y. et al., 2023), a ROS scavenger, suggesting a potential therapeutic strategy for alleviating neurocognitive dysfunction by targeting oxidative stress.

#### 3.3.2 Lysosomal disruption

Two mechanisms may be involved in the activation of inflammasome by lysosomal rupture after entosis of the particle matter: the release of cathepsin B (a lysosomal cysteine protease of the papain family) into the cytoplasm (Chu et al., 2009) and the triggering of K+ outflow (Hornung et al., 2008; Muñoz-Planillo et al., 2013). CA-074-Me, an inhibitor of cathepsin B and has been found in many studies to reduce NLRP3 activation by inhibiting the activity of cathepsin B. Besides, it has been shown that macrophages alone with the solute promoting agent Leu-Leu-O methyl ester can induce rapid K+ outflow prior to NLRP3 activation (Hornung et al., 2008). The above explanation for the release of cathepsin by damaged lysosomes, particularly cathepsin B, is a commonly proposed link, although how this further leads to K+ efflux is unclear (Amaral et al., 2018; Abdallah et al., 2011). However, recent studies have found that the plasma membrane damage caused by phagolysosome injury is a more important mechanism driving NLRP3 to activate aseptic inflammation (Beckwith et al., 2020). The plasma membrane damage is still being studied, and the stability of lysosome is still an important factor affecting the activation of NLRP3.

#### 3.3.3 Trans-Golgi network disruption

In addition, the breakdown of the trans-Golgi network activated NLRP3 into vesicles, which then recruit NLRP3 and facilitate the aggregation of ASCs, underscores the complex interplay between organelle dysfunction and NLRP3 inflammasome activation (Chen and Chen, 2018). This disintegration further exemplifies how organelle dysregulation can amplify inflammatory signaling pathways, contributing to the development of POCD.

In summary, the dysregulation of mitochondria, lysosomes, and the trans-Golgi network underlies the activation of the NLRP3 inflammasome in POCD, highlighting the intricate relationship between cellular organelles and inflammation. These insights offer promising avenues for the development of targeted interventions aimed at mitigating organelle dysfunction to treat or prevent POCD.

### 3.4 Downstream effects of NLRP3 inflammasome activation: pyroptosis and its role in POCD

**Figure 2:**
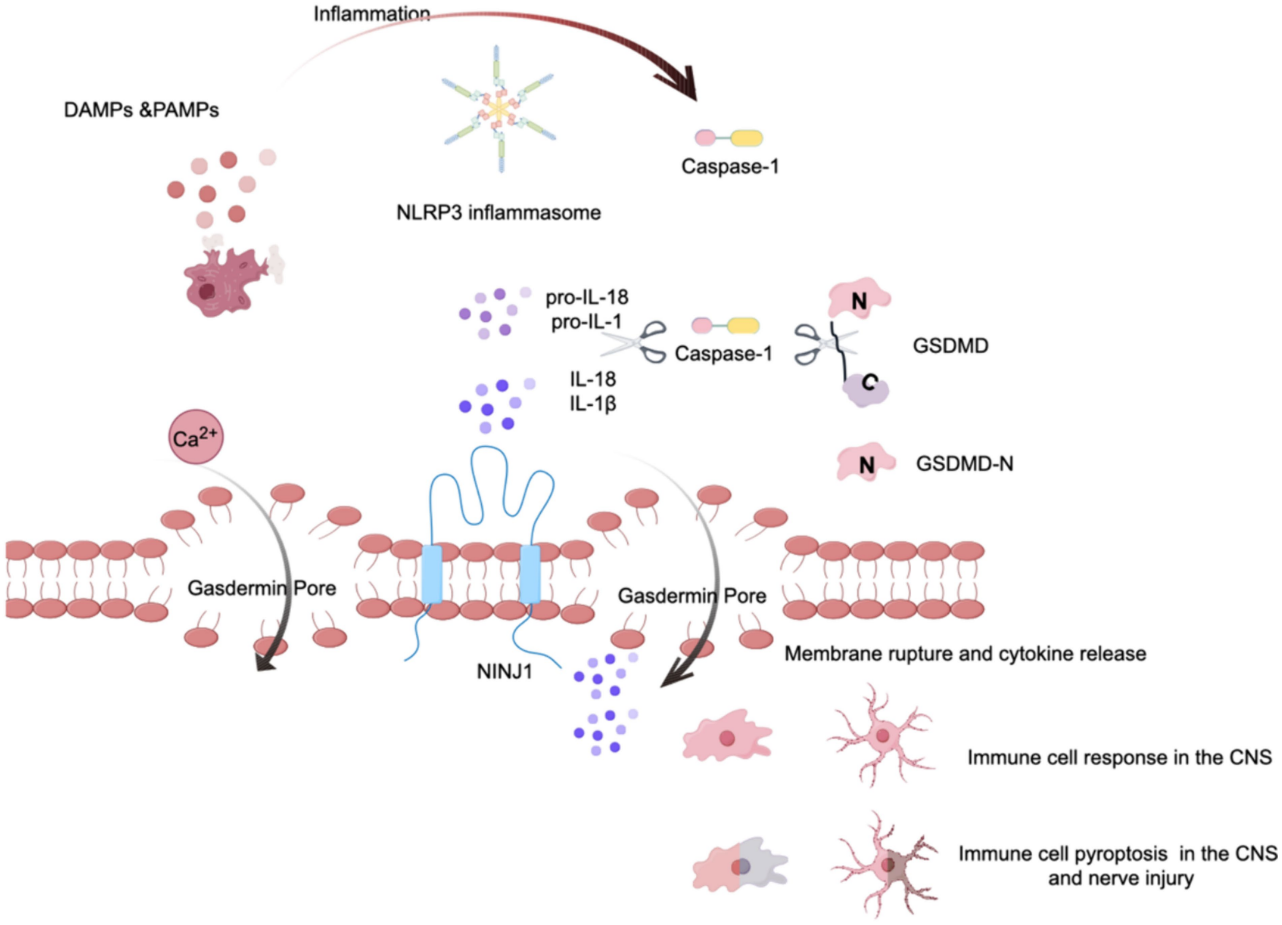
The role of pyroptosis via the NLRP3 inflammasome. This figure illustrates the cascade of events leading to pyroptosis mediated by the NLRP3 inflammasome. Initially, some stimulu trigger the assembly of the NLRP3 inflammasome, which then activates pro-caspase-1, converting it into its mature form, caspase-1. Acting like molecular scissors, activated caspase-1 cleaves gasdermin D (GSDMD), exposing its N-terminal domain. Concurrently, caspase-1 processes the proinflammatory cytokines pro-IL-1β and pro-IL-18 into their mature forms. The cleaved N-terminal fragment of GSDMD then binds to the plasma membrane and forms large, death-inducing pores, typically ranging from 10 to 20 nanometers in diameter. Additionally, cell surface proteins such as Ninjurin 1 (NINJ1) are involved in the rupture of the plasma membrane. This process culminates in the release of cytokines and calcium ions, leading to the pyroptosis of immune cells.

Pyroptosis is a critical downstream event triggered by the NLRP3 inflammasome (Zhou and Abbott, 2021), representing a form of programmed cell death that is highly inflammatory and characterized by cell swelling and rupture of the plasma membrane (Ding et al., 2019). Central to this process is the GSDMD protein, which serves as the executor of pyroptosis. GSDMD is characterized by two distinct structural domains: the N-terminal domain, which is responsible for pore formation, and the C-terminal domain, which regulates protein activity (Yao et al., 2024). The activation of GSDMD is contingent upon cleavage by activated caspase-1 (Yao et al., 2024), which separates the pore-forming N-terminal domain from the C-terminal autoinhibitory domain. Following this cleavage, the GSDMD N domain relocates to the plasma membrane, where it oligomerizes to form large pores inducing pyroptosis. This pore formation facilitates the release of proinflammatory cytokines such as IL-1β and IL-18 and promotes the flux of ions such as calcium and potassium across the plasma membrane (Aglietti et al., 2016; Ding et al., 2016). The cell damage caused by pyroptosis leads to reactivation of the NLRP3 inflammasome, amplifying the impact of the inflammasome response to cellular threats. Research has demonstrated that microglial pyroptosis in the hippocampus mediates sevoflurane-induced cognitive impairment in aged mice via the ROS-NLRP3 inflammasome pathway (Zhou Y. et al., 2023). Furthermore, studies have shown that a deficiency in GSDMD results in a marked reduction in IL-1β release following inflammasome activation (Broz et al., 2020). Given its fundamental role in inflammasome signaling, targeting GSDMD to control pyroptosis and the subsequent release of proinflammatory cellular contents represents a novel approach for mitigating POCD (see Figure 2).

### 3.5 Autophagy: an indispensable negative regulator of the NLRP3 pathway in POCD

**Figure 3:**
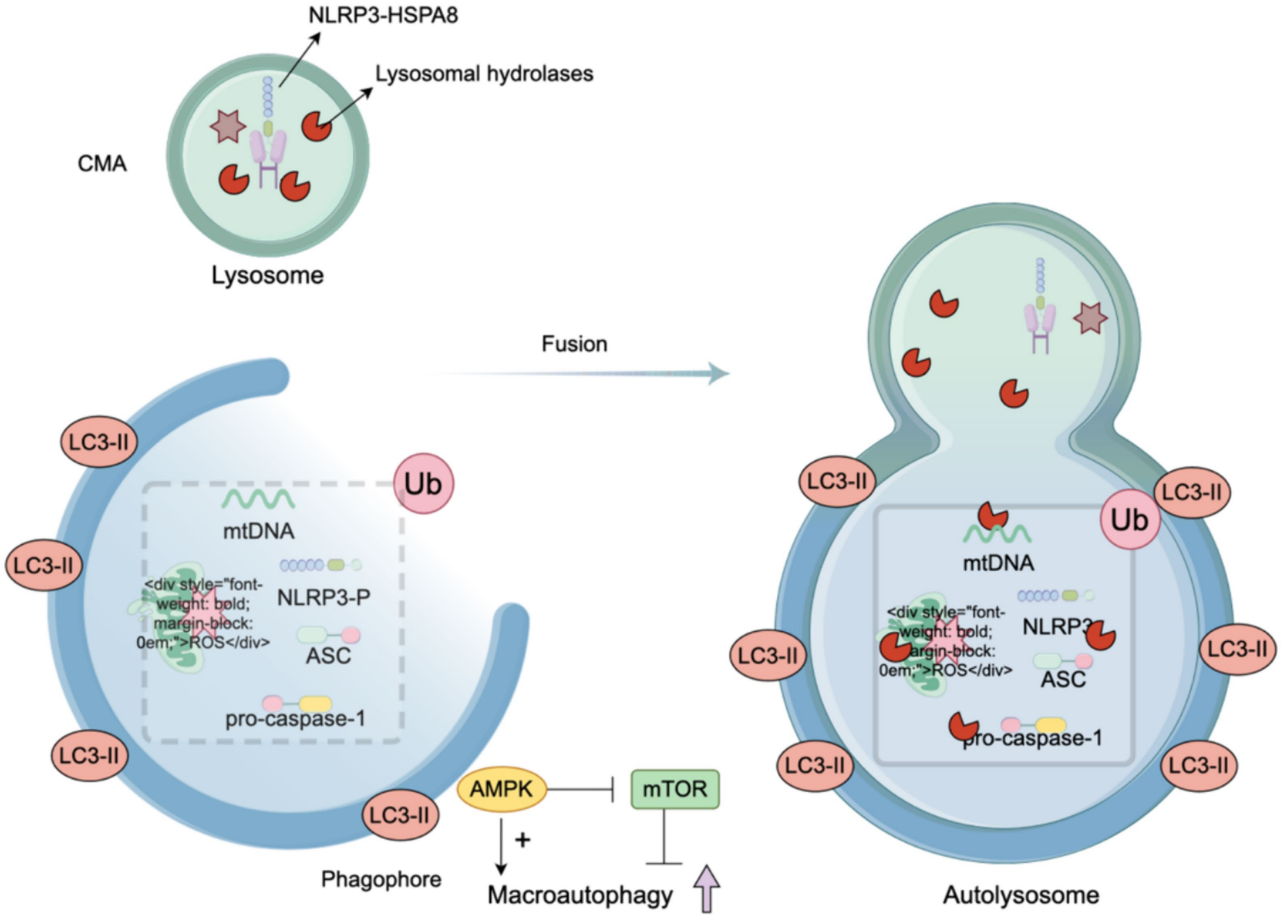
Autophagy, a regulator of the NLRP3. When the NLRP3 inflammasome is activated by various stimuli, autophagy is concurrently triggered. Additionally, autophagy is intricately regulated: AMP-activated protein kinase (AMPK) promotes autophagy, while the mammalian target of rapamycin (mTOR) signaling pathway negatively regulates autophagy. In response to activation, macrophages can directly inhibit the synthesis of the cNLRP3 inflammasome by degrading its assembly components, increasing NLRP3 phosphorylation, scavenging reactive oxygen species (ROS), and removing damaged mitochondria. The integration of LC3-II facilitates the elongation and closure of the phagophore membrane to form an autophagosome. The stimuli that activate the NLRP3 inflammasome are transported to lysosomes for degradation through mechanisms such as endocytosis and phagocytosis. The phagophore eventually fuses with the lysosome to form an autolysosome, where the phagocytic substances are digested or degraded.

Autophagy is a fundamental self-protection mechanism that involves the degradation of dysfunctional organelles and the removal of abnormal proteins, and it plays a crucial role in maintaining cellular integrity and function (Fleming et al., 2022). The relevance of autophagy extends to the repair mechanisms of the nervous system, notably through the degradation of the NLRP3 inflammasome and the removal of substances that cause NLRP3 inflammasome activation [such as damaged mitochondria and mtROS (Yan et al., 2019; Zhou J. et al., 2023)], thereby limiting overactivation of the NLRP3 inflammasome to negatively regulate the NLRP3 pathway (see Figure 3).

Autophagy is also subject to regulation by various substances. AMP-activated protein kinase (AMPK) promotes autophagy, whereas the mammalian target of rapamycin (mTOR) signaling pathway inhibits authophagy (Bonam et al., 2024). The integration of LC3-II contributes to the elongation and closure of the phagophore membrane to form an autophagosome (Huang et al., 2018). Some activators of the NLRP3 inflammasome are transported for lysosomal degradation by endocytosis and phagocytosis. The phagophore fuses with the lysosome to form an autolysosome, in which the phagocytic substance is digested or degraded.

**Figure 4:**
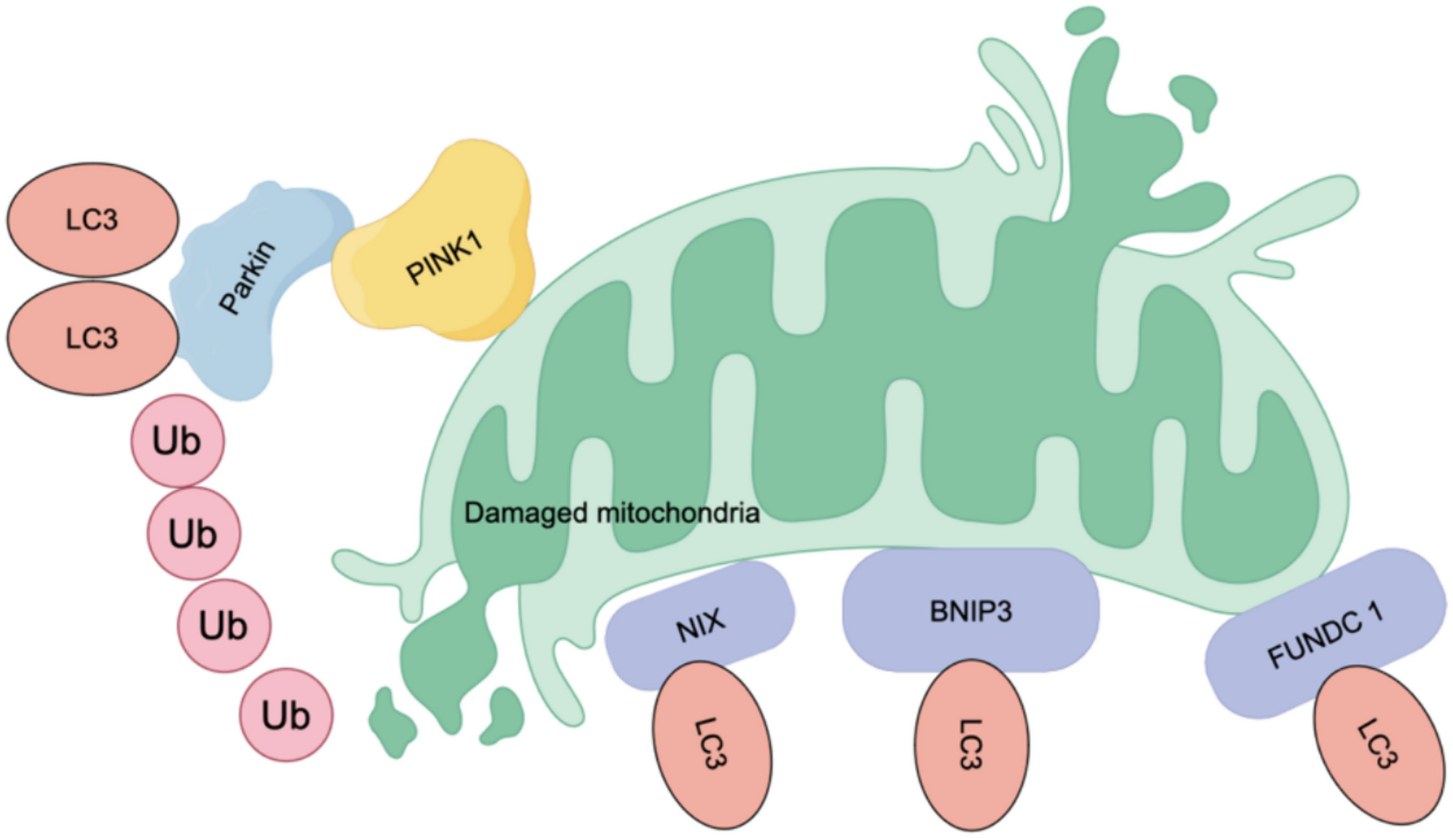
Mechanism of mitophagy. Mitophagy is mediated by ubiquitin or receptors. Ubiquitin mainly involves PINK (a serine/threonine-protein kinase)/Parkin (an E3 ubiquitin ligase). In damaged mitochondria, PINK1 fails to be transported to the inner mitochondrial membrane, resulting in the accumulation of PINK1 dimers on the outer mitochondria and the recruitment of Parkin, which activates Parkin and subsequently induces mitophagy. The second pathway is mediated by receptors, including NIX/BNIP 3 L, BNIP 3 and FUNDC 1, which are expressed on the outer membrane of mitochondria. These receptors share a common feature: they all contain LIR (LC3-interacting region) domains, which are crucial for their interaction with LC3 and the initiation of mitophagy. When dephosphorylated, FUNDC 1 shows a high affinity for LC3, enhancing mitophagy. Conversely, BNIP3 and NIX promote mitochondrial autophagy by stabilizing their interactions with LC3 through phosphorylation.

In addition, the selective elimination of abnormal mitochondria in the autophagy pathway, known as mitophagy, can target the removal of upstream events, leading to NLRP3 inflammasome activation (e.g., damaged mitochondria, mtROS). Mitophagy is mediated by ubiquitin or receptors. Ubiquitin-related mitophagy involves PINK1 (a serine/threonine-protein kinase)/Parkin (an E3 ubiquitin ligase) (Gao et al., 2015). In damaged mitochondria, PINK1 fails to be transported to the inner mitochondrial membrane, resulting in the accumulation of PINK1 dimers on the outer mitochondria and the recruitment of Parkin, which subsequently activates Parkin and induces mitophagy (Aerts et al., 2015; Gladkova et al., 2018). The second pathway is mediated by receptors including NIX/BNIP 3 L (Sandoval et al., 2008), BNIP 3 and FUNDC 1 (Chen et al., 2016). These receptors are expressed on the outer mitochondrial membrane and all contain LIR domains for interacting with the LC3 region, which are essential for initiating mitophagy (Li et al., 2023). Dephosphorylated FUNDC1 binds LC3 with high affinity to promote mitophagy (Liu et al., 2012), while BNIP3 and NIX promote mitophagy by stabilizing their interactions with LC3 through phosphorylation (Zhu et al., 2013; Rogov et al., 2017) (see Figure 4).

In the context of POCD, autophagy has emerged as a key protective mechanism. In sevoflurane anesthesia or surgically induced rat POCD models, the expression of AMPKα1, an important regulator of autophagy, was significantly downregulated (Yan et al., 2019). The activation of autophagy, achieved through the overexpression of AMPKα1, has been shown to alleviate cognitive impairments associated with surgery and anesthesia. This therapeutic effect is marked by an increase in the expression of autophagy markers such as LC-3-II and Beclin1 in the hippocampus and a decrease in p62 expression, indicating enhanced autophagic activity (Yan et al., 2019). Furthermore, recent studies have demonstrated that the inhibition of autophagy, characterized by a reduction in the LC3-II/I ratio and an increase in p62, is correlated with heightened activation of the NLRP3 inflammasome and exacerbated neuronal damage. The application of rapamycin, an autophagy activator, has been effective in reversing these adverse outcomes (Zhou J. et al., 2023), underscoring the potential of enhancing autophagy as a strategy for treating POCD. Sevoflurane significantly increases the levels of intracellular and mitochondrial ROS by 3.3-fold and 11.8-fold, respectively, which is accompanied by the accumulation of LC3B II/I and P62, decreasing mitophagy (Zheng et al., 2023). The use of an autophagy activator not only reduces reactive oxygen species (ROS) levels in cells and mitochondria but also blocks the elevated levels of LC3B II/I and P62, restoring mitophagy flux to normal levels (Zheng et al., 2023). These findings highlight the significance of autophagy in the prevention and treatment of POCD, suggesting that interventions aimed at promoting autophagic pathways could offer a promising avenue for mitigating the cognitive impairments associated with surgical interventions and anesthesia exposure (Chen et al., 2020).

### 3.6 Association of microglia with NLRP3 activation, autophagy and pyroptosis in POCD

**Figure 5:**
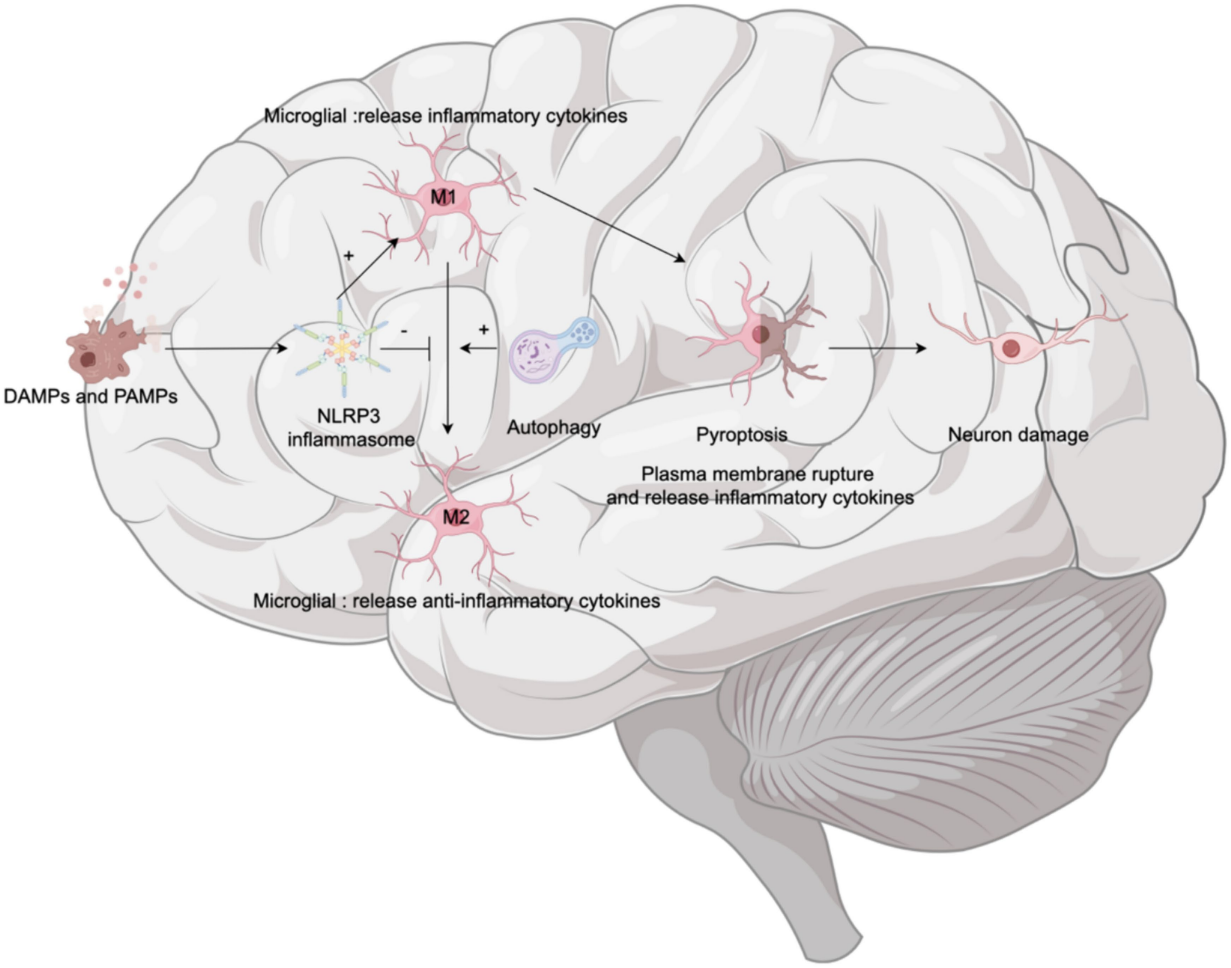
Microglia and cognitive dysfunction. Upon the detection of damage-associated molecular patterns (DAMPs) and pathogen-associated molecular patterns (PAMPs), the NLRP3 inflammasome within microglia is activated. This activation prompts microglia to polarize toward the proinflammatory M1 phenotype. Polarization to M1 microglia leads to breakdown of their plasma membrane—a process known as pyroptosis—which results in the release of inflammatory factors and subsequent neuronal damage. Concurrently, autophagy plays a crucial role by promoting the conversion of microglia from the M1 phenotype to the anti-inflammatory M2 phenotype, thereby alleviating neuroinflammation and potentially moderating cognitive decline.

Microglia, accounting for 10–15% of all brain cells (Dresselhaus and Meffert, 2019), are resident immune cells in the CNS that participate in CNS development, maintenance and response to damage and infection (Kent and Miron, 2024). In their resting state, microglia monitor their environment; upon activation, they transform into one of two polarization phenotypes depending on the activation state: the proinflammatory M1 phenotype (neurotoxic effects) or the anti-inflammatory M2 phenotype (neuroprotective effects). M1 microglial release proinflammatory cytokines (such as IL-1b, TNF-a, IL-18, and IL-6), chemokines, and reactive oxygen species (ROS), resulting in neuronal cell injury and BBB disruption (Brown and Vilalta, 2015). M2 microglia release anti-inflammatory cytokines IL-10, arginase (Arg-1) and chitinase-3 (Chil3) to maintain and repair neural tissue (Kanazawa et al., 2017) (see Figure 5).

The NLRP3 inflammasome is abundantly expressed in microglia. Activation of the NLRP3 inflammasome also influences the phenotypic transformation of microglia. Elevated NLRP3 levels are associated with the overexpression of the M1 microglial marker CD86, and the relative suppression of M2 microglial marker CD163 immunoreactivity (Ibrahim et al., 2023). For example, in models of Alzheimer’s disease in mice, overactive NLRP3 drives microglia to polarize toward the inflammatory M1 phenotype, which is characterized by upregulated caspase-1 and IL-1β, contributing to impaired spatial memory function (Ibrahim et al., 2023). These effects can be mitigated by deficiencies in NLRP3 or caspase-1 (Heneka et al., 2013). Furthermore, microglial autophagy not only inhibits NLRP3 activation as a negative regulator but also transforms microglia from a proinflammatory state (M1) to a favorable anti-inflammatory state (M2) (Wu et al., 2021), thus playing a protective role in the nervous system. However, microglial pyroptosis in the hippocampus mediates sevoflurane-induced cognitive impairment in aged mice (Zhou Y. et al., 2023). Therefore, transforming the microglial phenotype from the proinflammatory M1 phenotype to the anti-inflammatory M2 phenotype is essential and represents a therapeutic approach for neurodegenerative diseases (Yao and Zu, 2020), including POCD.

In summary, when exposed to endogenous and exogenous stimuli, the NLRP3 inflammasome in microglia is activated, promoting the M1 phenotype and enhancing pyroptosis—a downstream event that leads to cell death and neuroinflammation, thus exacerbating the progression of neurodegenerative diseases. Autophagy serves as a countermeasure, mitigating cognitive deterioration by clearing inflammasome activators, reducing proinflammatory substances, and promoting the polaruzation of microglia toward the M2 phenotype.

### 3.7 Neurotransmitters

Neurotransmitters are chemicals that promote neuronal communication and play an important role in synaptic plasticity, which is closely related to learning and memory, cognitive function, and brain injury recovery (Yang et al., 2023). Among neurotransmitters, gamma-aminobutyric acid (GABA) is the most important inhibitory transmitter in the mammalian brain (Samakashvili et al., 2011; Farrant and Nusser, 2005).Multiple types of GABA are present in the cerebrospinal fluid of AD patients (Samakashvili et al., 2011; Farrant and Nusser, 2005), and Wang L.-Y. et al. (2023) also found abnormal increases of GABA in the hippocampus of surgically exposed mice. Interestingly, the GABAergic system could promote macrophage mediated NLRP3 inflammasome activation (Krabbe et al., 2018). Lu-Ying Wang knocked out the astrocyte specific NLRP3 gene in mice and found that it could reverse TF-induced impulsive and cognitively impaired behavior, reduce GABA levels in cells, ameliorate NLRP3-related inflammatory responses, and restore hippocampal neuronal degeneration (Wang L.-Y. et al., 2023).

## 4 Treatment

We collected nearly 3 years of research on NLRP3 related drugs used to alleviate POCD, and the results are presented in Table 1.

**Table 1:** NLRP3 inflammasome-associated drugs.

Drug	Functional mechanism or site	Effect	References
Melatonin	Reduce the phosphorylation of nuclear factor NF-κB and inhibit its nuclear translocation	NF-κB, NLRP3, ASC, caspase-1, and IL-1β ↓	Zhu et al. (2023)
Annexin-A1 Tripeptide	Inhibit postoperative NLRP3 inflammasome activation.	ASC, NLRP3, and IL-1β ↓	Zhang et al. (2022)
Vitamin D3	The inactivation of the NLRP3 inflammasome	NLRP3, IL-1β, and IL-18 ↓	Jiang J. et al. (2022)
NU 9056	Inhibits the acetylation of NLRP3 (inhibits NLRP3 oligomerization without affecting its recruitment to dispersed trans-Golgi network (dTGN))	caspase-1, and IL-1β ↓	Zhang Y. et al. (2023)
Dexmedetomidine	Regulating NLRP3 and ASC expression, inhibiting NF-κB	NLRP 3, ASC, caspase-1, and IL-1β ↓	Eskandr et al. (2018); Godlewski and Castellanos (2018)
Helenine	Interrupt the formation of the NLRP3-NEK7 interaction to block the inflammasome assemblage and activation	caspase-1, IL-1β ↓	Fang et al. (2024)
The ethyl acetate fraction of Bungeanum	Inhibiting the activation of NLRP3 inflammasomes and GSDMD-mediated pyroptosis	NLRP3, caspase-1, GSDMD-N, IL-1β, and IL-18 ↓	Zhao M. et al. (2021)
Electroacupuncture	Inhibit the activation of NLRP3 inflammasome and NF-κB	the phosphorylation of IκB-*α*, and NLRP3 ↓	Sun et al. (2022); Wang W. et al. (2024)
Ac-YVAD-cmk	An inhibitor of caspase-1 and mitigate mitochondria dysfunction	caspase-1, NLRP3, IL-1β, and IL-18 ↓	Zheng et al. (2023)
Bergapten	Inhibits NLRP3 inflammasome activation and pyroptosis via promoting mitophagy	caspase-1, IL-1β, ASC, GSDMD ↓	Luo et al. (2023)
Scoparone	Suppresses mitophagy-mediated NLRP3 inflammasome activation	caspase-1, GSDMD, IL-1β, and ASC ↓	Ibrahim et al. (2023); Feng et al. (2023)
Polygala saponins	Via SHP-2-Mediated mitophagy	NLRP3 ↓	Qiu et al. (2022)
Rapamycin	Activate autophagy resulted in the inhibition of NLRP3 inflammasomes	NLRP3, Caspase-1, IL-1β and IL-18 ↓	Zhou J. et al. (2023); Bonam et al. (2024)
Olaparib	Activate autophagy resulted in the inhibition of NLRP3 inflammasomes	NLRP3, ASC, and IL-1β ↓	Lu et al. (2024)
TREM2	Regulates mitophagy and NLRP3 inflammasome	NLRP3, and IL-1β ↓	Jiang W. et al. (2022)
N-acetylcysteine (NAC)	A ROS scavenger	GSDMD-N, IL-1β, IL-18, NLRP3, ASC, and cleaved-caspase-1 ↓	Zhou Y. et al. (2023)
JWH133	The CB2R agonist-CB2R promoted ubiquitination and autophagy-induced degradation of NLRP3	NLRP3 ↓	Tiberi et al. (2022)
zDHHC12	Targets NLRP3 for palmitoylation to promoting NLRP3 degradation through the chaperone-mediated autophagy (CMA) pathway.	NLRP3 ↓	Wang L. et al. (2023)
Necrosulfonamide (NSA)	A pyroptosis inhibitor	GSDMD-N, cleaved caspase-1, ASC, IL-1β and IL-18 ↓	Fan et al. (2018)
Punicalagin	A pyroptosis inhibitor by impariming membrane permeability/ antioxidant	GSDMD-N, caspase-1, IL-1β, and ROS ↓	Rathkey et al. (2018); Martín-Sánchez et al. (2016)
DUSP14	Inhibit NLRP3 inflammasome-dependent pyroptosis	GSDMD-N, cleaved caspase-1, IL-1β and IL-18 ↓	Que et al. (2020)
ACY-125	HDAC6 inhibitor, HDAC6 mediate microglial pyroptosis by regulating NLRP3	NLRP3, IL-1β, and GSDMD-N ↓	Lin et al. (2024)
VX765	Inhibited caspase-1 suppressed the expressions of GSDMD and its cleavage form GSDMD-NT, and reduced pyroptosis in brain	GSDMD-N, IL-1β, and TNF-α ↓	Piippo et al. (2018)
Intermittent theta burst stimulation	inhibiting neuronal pyroptosis and regulating microglial polarization	Caspase1, IL-1β, IL-18, ASC, and GSDMD ↓	Zhang Y. et al. (2023)

### 4.1 Strategies for reducing NLRP3 inflammasome activation to preserve cognitive function after surgery

The NLRP3 inflammasome is a pivotal component in the development of POCD, making reducing of its activation and formation a critical aspect of POCD prevention and treatment. Various substances and methods have demonstrated potential in targeting the NLRP3 inflammasome, offering promising avenues for safeguarding cognitive function postsurgery.

Melatonin, known for its antioxidant properties and ability to protective agent against mitochondrial damage, plays a significant role in modulating inflammatory responses. It specifically targets the NLRP3 inflammasome and modulates the NF-κB pathway, which includes altering NF-κB phosphorylation, H3 histone acetylation, and NF-κB nucleation. These actions contribute to protecting against cognitive impairments in aged models (Zhu et al., 2023). The protein annexin-A1 Tripeptide (ANXA1), which is regulated by glucocorticoids, reduces the activation of the NLRP3 inflammasome across ages after surgery and improves cognitive dysfunction induced by neuroinflammation (Zhang et al., 2022). Similarly, vitamin D3 protects against POCD by deactivating the NLRP3 inflammasome and reducing the release of proinflammatory cytokines (Jiang J. et al., 2022). Lysine acetyltransferase 5 (KAT5) (Zhang Y. et al., 2023) assists in NLRP3 acetylation, promoting the oligomerization of NLRP3 and the subsequent assembly of the inflammasome, whereas NU 9056 blocks this process, offering the potential to treat NLRP3-associated diseases (Zhang Y. et al., 2023). Dexmedetomidine (DEX) is a widely used sedative agent in the clinic that mitigates the occurrence of POCD through multiple mechanisms, including inhibiting NF-kB, inhibiting NLRP3 inflammasome activation, regulating ASC expression, and promoting NLRP3 inflammasome degradation (Cho et al., 2022; Zeng et al., 2023). The components and techniques derived from traditional Chinese medicine relieve POCD. Helenine inhibits NLRP3-dependent ASC oligomerization and hinders the binding of NEK7 to NLRP3 (Fang et al., 2024), making it a promising drug for targeting the activation of the NLRP3 inflammasome. The ethyl acetate fraction of Bungeanum suppresses both the activation of the NLRP3 inflammasome and GSDMD-mediated pyroptosis in aging mice to ameliorate cognitive deficits (Zhao M. et al., 2021). Electroacupuncture (EA), an alternative acupuncture technique, suppresses the activation of the NLRP3 inflammasome via the inhibition of the NF-κB pathway, which is a potential technique for alleviating POCD (Sun et al., 2022; Wang W. et al., 2024).

These diverse strategies underline the importance of targeting the NLRP3 inflammasome as part of a comprehensive approach to prevent or treat POCD. By mitigating NLRP3 activation, it is possible to protect against cognitive decline associated with surgical procedures, improving the quality of life of patients undergoing surgery.

### 4.2 Autophagy is appropriately enhanced to preserve cognitive function

Autophagy, a critical cellular housekeeping mechanism, plays a vital role in removing damaged organelles and degrading aberrant macromolecular proteins. As a regulatory mechanism of NLRP3, autophagy limits the activation of the NLRP3 inflammasome by scavenging endogenous inflammasome activators, such as mtDNA, reactive oxygen species (ROS), dysfunctional mitochondria, inflammatory components, and cytokines (Biasizzo and Kopitar-Jerala, 2020; Pradel et al., 2020; Zhao S. et al., 2021). Furthermore, the role of mitochondria as a platform for NLRP3 and ASC recruitment (Qiu et al., 2022) underscores the importance of mitophagy in maintaining mitochondrial integrity and regulating NLRP3 activation.

Studies have illuminated the protective role of autophagy against cognitive impairments induced by anesthesia or surgery. For instance, Yeru Chen et al. demonstrated that mitophagy impairment is involved in sevoflurane-induced cognitive dysfunction in aged rats (Chen et al., 2020). The use of Ac-YVAD-cmk, a specific caspase-1 inhibitor, has been shown to block caspase-1-dependent mitochondrial autophagy, reducing lipid peroxidation and mitochondrial ROS accumulation and alleviating sevoflurane-induced POCD (Zheng et al., 2023). Compounds such as Bergapten (BeG), a furocoumarin phytohormone with anti-inflammatory properties, have been found to suppress NLRP3 inflammasome activation and pyroptosis by promoting mitophagy and maintaining mitochondrial homeostasis (Luo et al., 2023). More specifically, BeG is involved in the regulation of mitochondrial gene expression and reactive oxygen metabolism in macrophages (Luo et al., 2023). Similarly, scoparone has been reported to enhance mitophagy, and mitigate reactive oxygen species (ROS) production and NLRP3 inflammasome activation, reducing inflammation (Feng et al., 2023). Scoparone also promoted the polarization of microglia from the M1 to the M2 phenotype (Ibrahim et al., 2023), which is involved in the protection of cognitive function. Polygala saponins inhibit NLRP3 inflammasome-mediated neuroinflammation via SHP-2-mediated mitophagy (Qiu et al., 2022). In addition, other studies have reported similar effects of drugs such as rapamycin (Zhou J. et al., 2023; Bonam et al., 2024), olaparib (Lu et al., 2024), TREM2 (Lu et al., 2024) and N-acetylcysteine (NAC) (Zhou Y. et al., 2023). Endocannabinoid receptor subtype 2 (CB2R) is a receptor involved in central nervous system immune regulation and plays a protective role in neurodegenerative diseases (Tiberi et al., 2022). JWH133, the CB2R agonist CB2R, promotes ubiquitination and autophagy-induced degradation of NLRP3 (Jiang F. et al., 2022). zDHHC12 is a member of the zinc-containing finger DHHC (Asp-His-His-Cys) type (zDHHC) palmitoyl S-acyltransferase (pat) family, which targets NLRP3 for palmitoylation, thereby promoting NLRP3 degradation through the chaperone-mediated autophagy (CMA) pathway (Wang L. et al., 2023).

In conclusion, enhancing autophagy represents a strategic approach to protect cognitive function following surgery and anesthesia by directly targeting the NLRP3 inflammasome and its upstream activators. The diverse range of compounds and mechanisms discussed underscore the potential for developing targeted therapies to prevent or ameliorate POCD.

### 4.3 Targeting GSDMD-induced pyroptosis to combat POCD

GSDMD-induced pyroptosis, an inflammatory programmed cell death pathway, has been identified as a contributing factor to the cognitive impairments observed after the administration of volatile anesthetics such as isoflurane in aged mice (Fan et al., 2018). Addressing this pathway offers a promising strategy for alleviating postoperative cognitive dysfunction (POCD). GSDMD-induced pyroptosis, a novel inflammatory form of programmed cell death, is associated with cognitive dysfunction induced by the volatile anesthetic isoflurane in aged mice (Fan et al., 2018).

Necrosulfonamide (NSA), a chemical inhibitor that specifically targets GSDMD, prevents the formation of pyroptotic pores, thereby inhibiting pyroptosis (Rathkey et al., 2018). This mechanism has shown potential in mitigating neurological impairments induced by sevoflurane, suggesting a therapeutic approach for POCD through the pharmacological blockade of pyroptosis (Zhou Y. et al., 2023). Similarly, punicalagin, a complex natural polyphenol, acts as a pyroptosis inhibitor by impairing inflammasome-driven membrane permeability without affecting caspase-1 activity or the processing of IL-1β and GSDMD (Martín-Sánchez et al., 2016; Torre-Minguela et al., 2021). Its role in modifying plasma membrane fluidity can disrupt the assembly of N-GSDMD into the membrane, highlighting an additional avenue for pyroptosis inhibition (Torre-Minguela et al., 2021). Moreover, the antioxidant properties of punicalagin may further reduce pyroptosis by influencing the role of reactive oxygen species (ROS) in promoting N-GSDMD pore formation (Evavold et al., 2021). DUSP14, a regulatory protein, inhibits NLRP3 inflammasome-dependent pyroptosis, potentially serving as a critical mechanism for reducing neuronal injury and cognitive impairment in elderly rats (Que et al., 2020). After deacetylation, heat shock protein 90 (HSP90) has an enhanced protein binding ability and can bind to the NLRP3 protein to prevent its degradation (Piippo et al., 2018). The ability of ACY-125 to inhibit the deacetylation of HSP90 further demonstrates its potential to regulate microglial pyrosis by inhibiting NLRP3 at the molecular level (Lin et al., 2024). VX765 acts as a caspase-1 inhibitor that suppresses the expression of GSDMD and its cleaved form GSDMD-NT and reduces pyroptosis (Xu et al., 2019). Intermittent theta-burst stimulation in cerebral ischemic mice could improve nerve function by inhibiting neuronal pyroptosis and regulating microglial polarization via the NFκB/NLRP3 signaling pathway (Luo et al., 2022).

In summary, targeting GSDMD-induced pyroptosis is a multifaceted approach for mitigating POCD involving the inhibition of pyroptotic pore formation, the modulation of membrane permeability, and the regulation of inflammatory responses. These strategies highlight the importance of understanding cellular death mechanisms in developing effective treatments for POCD.

## 5 The current dilemma of POCD

### 5.1 POCD animal model

Rodents are the most common animal models for the study of postoperative cognitive dysfunction, and the establishment of such models involves two categories: simple anesthetic drug induction and complex surgical trauma. In addition, based on susceptibility to postoperative cognitive dysfunction, the rodent model also considers age, genetics, and preoperatively related cognitive dysfunction. There are also some relatively well-developed methods for assessing cognitive dysfunction in rodents, such as the fear condition test, the open field test, the Morris Water Maze test, the Y Maze test, and so on.

Through numerous animal experiments, scientists have identified a close relationship between the NLRP3 inflammasome pathway and POCD. This is since rodents share some similarities with humans in the NLRP3 inflammasome pathway. The NLRP3 inflammasome plays a key role in antimicrobial defense as well as in inflammatory diseases. In both mice and humans, NLRP3 is a highly sensitive but non-specific pattern recognition receptor that responds to perturbations of cellular homeostasis through a range of different stimuli (Sharma and Kanneganti, 2021). Activation of the NLRP3 inflammasome can be activated by stimuli including melanotin, uric acid crystals, beta-amyloid fibril, and extracellular ATP. The process is similar in mice and humans. Although the NLRP3 inflammasome in mice and humans is similar in basic structure and function, there may be differences in specific activation mechanisms. For example, K+ ion outflow is thought to be a common feature of many NLRP3 inflammasome (Xia et al., 2018), but in humans, it has also been reported that NLRP3 activation does not depend on K+ ion outflow (Schmacke et al., 2022). In addition, NEK7, as a mitotic kinase, allows interphase assembly and activation of the NLRP3 inflammasome in both humans and rodents (Sharif et al., 2019; Xiao et al., 2023), but their mechanisms may differ depending on whether this is achieved through activation of the K channel.

In summary, the NLRP3 inflammasome in rodents and humans is similar in basic function and structure, but there may be differences in specific activation mechanisms and interactions. These differences may result from physiological and metabolic differences between species, as well as adaptations to different environmental factors during evolution. Due to species differences, critically evaluating inflammasome research in cognitive dysfunction, including POCD and Alzheimer’s disease and related disorders, will be critical. In other words, it calls for the development of animal models that better mimic humans.

### 5.2 POCD biomarkers

Bridging the gap between research and clinical applications, potential POCD biomarkers are something we need to focus on. The biomarkers of POCD are mainly divided into brain-derived biomarkers related to nerve injury and neurotoxicity and brain-derived biomarkers related to neuronutrition. Their functions are shown in Table 2.

**Table 2:** Biomarkers of postoperative cognitive dysfunction.

Biomarkers (Nerve injury/ neurotoxicity)	Function	Effect
Neurofilament light (NfL)	A subunit of neurofilament protein, a cylindrical protein in the cytoplasm of neurons (Halaas et al., 2018)	NfL was elevated in cerebrospinal fluid and plasma in POCD
p⁃Tau protein	Tau protein: a component of the microtubules of neurons; p⁃Tau protein damages the integrity of axonal microtubules (Buchholz and Zempel, 2024)	Plasma Tau protein was significantly associated with the incidence and severity of POCD (Ballweg et al., 2021)
Phosphorylated neurofilament heavy subunit-H (pNF-H)	One of the skeleton proteins of neurons of the central nervous system; Reflecting the damage of structural neurons (Zhang et al., 2019)	An independent risk factor in POCD (Inoue et al., 2017)
Neuron-specific enolase (NSE)	A glycolytic enzyme found in neurons and endocrine cells	Predicting the occurrence and severity of POCD (Mietani et al., 2021)
β-amyloid (Aβ)	The product of amyloidosis in neurons and glial cells	Cerebrospinal fluid Aβ40 is an independent risk factor for POCD (Wang et al., 2022)
Matrix metalloproteinase 9 (MMP-9)	Degradation of extracellular matrix and disruption of tight connections to break down the blood–brain barrier	Serum MMP⁃9 level in POCD patients increased significantly (Xie et al., 2016)
Ubiquitin C terminal hydrolase-L1 (UCH-L1)	Highly specific neuronal proteins	Since UCH⁃L1 is not present from other non-neurological sources; Specific biomarkers of neuronal damage (Majewski et al., 2020)
Glial fibrillary acidic protein (GFAP)	The main intermediate filament protein of mature astrocytes	Serum GFAP levels reflect glial cell damage (Gailiušas et al., 2019)

Biomarkers (Neurotrophic)	Function	References
Progranulin PGRN	Neuronutrition, elongation of axon and proliferation of neural stem cells	PGRN in CSF was an independent protective factor for POCD (Wang et al., 2022)
Brain derived neurotrophic factor BDNF	Regulating synaptic plasticity and neurotransmission	The expression of BDNF in POCD aged mice decreased significantly (Xue et al., 2022)

There are few clinical trials or observational studies that provide direct clinical evidence for interventions such as the NLRP3 inflammasome or autophagy. Most of the studies were preclinical studies based on animal models and some circumstantial evidence. What we do know for sure is that some patients who have undergone anesthesia or surgery do show inflammation in their blood samples. In POCD patients who underwent total hip replacement, their inflammatory markers (including CRP, S-100B, IL-1β, IL-6, and TNF-*α*) were significantly elevated compared to non-POCD patients (Peng et al., 2013; Li et al., 2012), but these inflammatory factors were non-specific. Future studies should determine whether the NLRP3 inflammasome is involved in POCD in human patients.

### 5.3 Challenges and considerations in moving from preclinical studies to clinical trials

**Figure 6:**
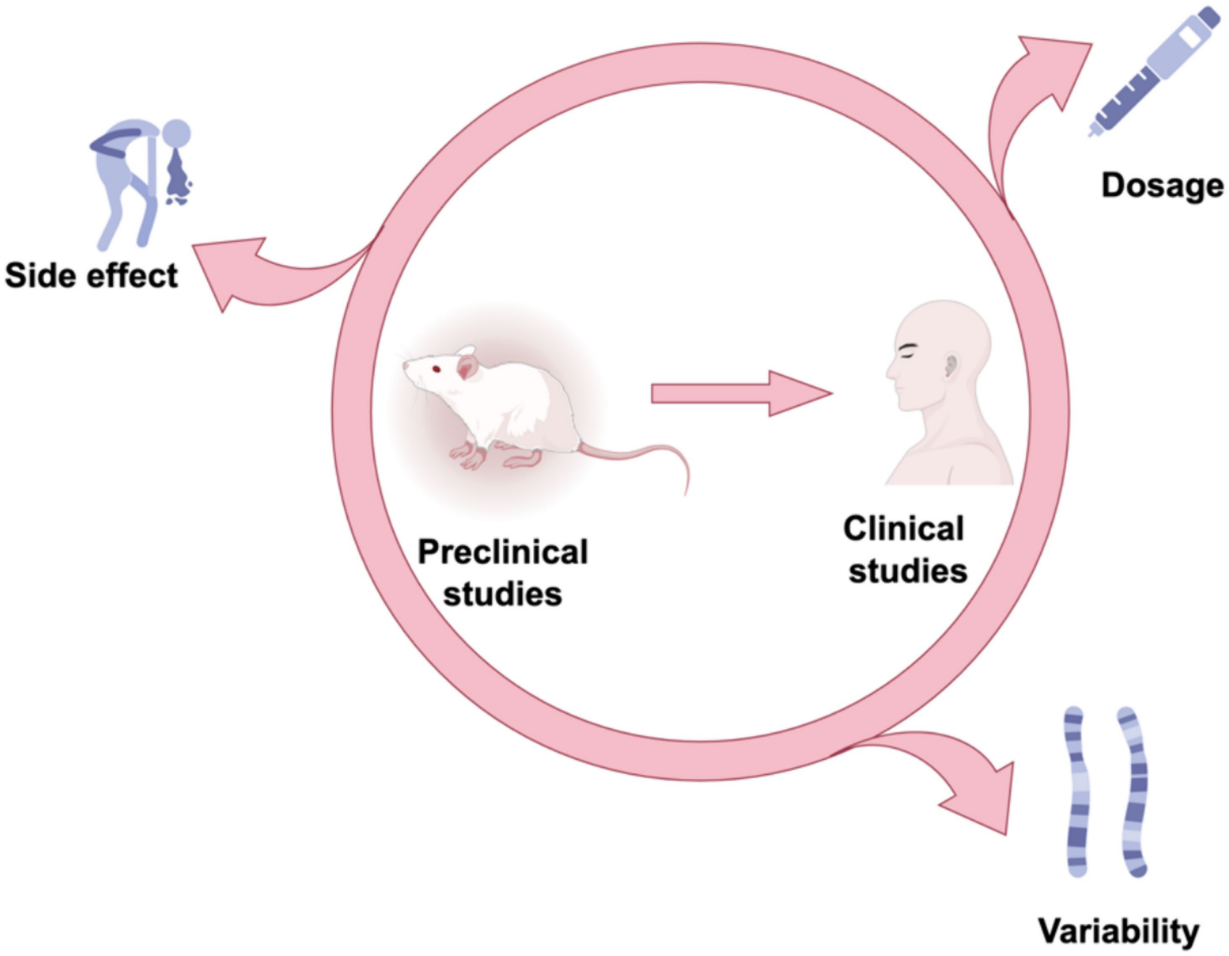
Future direction. To determine the future direction from preclinical to clinical studies, we need to focus on dosing issues, potential side effects, and patient variability.

In the process of advancing from preclinical research to clinical studies, ethical issues may be the most important problems we face in future research. Ethical concerns may relate to potential side effects, dosing issues, and patient variability (Figure 6). In preclinical studies, although a series of safety and efficacy evaluations have been conducted, after entering clinical trials, still needs to pay close attention to the potential side effects that may be brought by the drug, to ensure the safety of the subjects. Determining the appropriate drug dose is an important step in clinical trials. The pharmacokinetic properties of the drug need to be carefully studied, to ensure the efficacy and safety of the dose. Variability between patients may affect the efficacy and safety of the drug. In clinical trials, should fully consider the diversity of patient groups, and formulate corresponding research strategies.

## 6 Conclusion

NLRP3 inflammasome activation and its role in inducing pyroptosis are central to the development of postoperative cognitive dysfunction (POCD), and strategies targeting its inhibition, enhancing autophagy, and preventing pyroptosis show promise for protecting cognitive functions in surgical patients. Despite progress, significant knowledge gaps remain concerning the structure of the NLRP3 inflammasome, its activation mechanisms in the CNS, and its precise impact on POCD. Addressing these gaps is crucial for advancing targeted therapies. Emerging evidence suggests that modulating this pathway could offer novel approaches for mitigating the cognitive impairments associated with anesthesia and surgery, highlighting the need for further investigations into the intricate roles and regulatory mechanisms of the NLRP3 inflammasome within the central nervous system to uncover new therapeutic targets for POCD.

A series of compounds have been shown to alleviate cognitive dysfunction by regulating the upstream and downstream of the NLRP3 inflammasome, providing candidates for the protection of cognitive function. However, further research is needed to confirm whether these compounds are directly dependent on the NLRP3 inflammasome to play a role in alleviating cognitive dysfunction. In addition, it is necessary to test their specificity to the NLRP3 inflammasome and whether better treatment results can be achieved through the combination of multiple drugs.
